# Swaged vs. Cast Dentures

**Published:** 1917-10

**Authors:** 


### SWAGED vs. CAST DENTURES

New and improved methods of casting metals have resulted in the cast denture superseding, in a large measure, the older type of swaged bases. Some operators, however, still favor the swaged base, claiming that it is superior both as regards efficiency and economy. Basil Jones, D.D.S., has has considerable experience with both methods of preparing metallic bases, and recounts his opinion of them in an article published in the December issue of the *Commonwealth Dental Review.* This paper, aside from its particular interest for students of dental metallurgy, contains much of value for all prosthetic workers as well. The following features are recognized as being of advantage in the cast denture: (1) Time saved in construction. No sand moulds, discs and counter-dies are required, and the process is quicker and more direct in results. (2) A cast denture made from a good plaster impression will at once adapt itself to the tissues and at once shows excellent suction. In this way the operator can determine whether or not the completed denture is likely to be satisfactory. (3)

In partial cases, or those having severe undercuts, a cast base can be prepared with less difficulty than would be the case if dies and counter-dies were necessitated. (4) Working with a wax model, it is possible to secure a differentiation of thickness in the finished base. Obviously, great difficulty would be experienced in attempting this with a swaged base. (5) A cast base requires no solder for attachments, etc., thus eliminating the danger of warping and effecting a great saving of time. (6) When carving up the wax model it is possible to extend the denture in a manner calculated to do away with the necessity for band support. More perfect results are obtained in this way than would be the case if the same thing were attempted in swaged work. (7) In the case of a close bite, a cast denture may be made to carry all the articulating surface required for a confined space.

So much for the favorable features of cast dentures; now a few of their disadvantages, taking the cast gold base as an example: (1) In casting metals it is always necessary to use more material than is actually required for the completed case, consequently there is always the danger of wastage and the necessity of using an excessive amount of material. When making a swaged base, it is possible to obtain a pattern and with it secure the exact amount of material of a specified thickness, etc., required. In this way no surplus supply is needed. This is an important feature when precious metals are involved. (2) A cast denture requires more or less filing and finishing. This represents wastage of material. A cracking or giving of the investment material results in an undesirable thickness in certain portions of the completed case. And, again, the sprues must be removed and all traces of them obliterated by filing, etc. These operations must of necessity result in loss of material. (3) The certainty of an even distribution or uniform thickness of material is best secured by the swaging process than by any method of casting. Heated wax responds so readily to the slightest pressure that a thinning may occur without detection. When polishing the completed case these thin areas are readily exposed. The danger of such an experience when using metal of a uniform thickness is certainly not very great. (4) There is want of elasticity and tenacity in cast metals. This is due to the fact that in heating the alloy to a high temperature we lessen the tenacity of each of the constituent metals of the alloy and the benefit of such metals as copper and silver is lost. "Further, we lose the effect of annealing at a low temperature and rolling, which porduces density and elasticity by a perfect union of the alloyed metals. I will admit (says Dr. Jones) that I have seen cast dentures extremely tough and elastic, but they have not that constant and soft elasticity which is produced by the rolling and swaging of gold. With regard to this, I would instance the old Butterfly partial upper dentures made some twenty-five years ago and carrying some eight or ten teeth; these plates, after many years' hard wear, seem to have simply moulded themselves to the mouth; teeth adjoining the artificial ones may have been extracted and recession of the alveolar ridge taken place, yet the form and elasticity of the denture has given it a firm adaptation to the altered mouth; new teeth may even be added to fill the places of those lost by extraction without affecting the fit of the denture. Will such an experience be shown with our present cast dentures? To my mind, platinum added to a cast denture produces brittleness and reduces the elasticity of the cast. On the other hand, one grain of platinum added to one dwt. of twenty carat gold and rolled thoroughly and annealed gives the most elastic alloys." (5) If the wax pattern is uneven in thickness then the cast gold will contract unevenly during solidification and so result in an imperfect cast. Such a condition may result although the pressure used in casting is ample and evenly distributed. There is an added danger of the surface of the cast denture being richer in copper and silver crystals than the centre. Annealing and rolling would have an opposite tendency, viz., the softer metallic constituents of the alloy would be distributed evenly among the other parts of the alloyed mass. (6) More difficulty is experienced in repairing a cast denture than would be the case with a swaged one. Application of solder to a cracked cast denture usually results in a recurrence of the trouble just where the repair has been made. This is particularly noticeable in cases where the cast metal is thin: A swaged denture may be mended by burnishing pure gold and uniting the parts with solder. (7) Some metals hold in a marked degree the property of occluding gases while in a molten condition. On cooling, the metallic mass liberates these gases and a condition of ''spitting'' or ''vegetation'' is apparent on the surface of the solidified metal. Small holes or ''craters'' may be observed sometimes with the naked eye, and when examined with the aid of a magnifying glass the whole surface will present an appearance of porosity. Buffing and polishing may conceal these imperfections somewhat, but they are still there and afford excellent places for food attachment. A cast denture does not keep its color as readily as does the swaged because, as Dr. Jones claims, some of the alloyed metals, such as copper, may be forced to the surface and so come into intimate contact with the fluids of the oral cavity. This is a condition that is singularly absent in a swaged base. (8) There is a greater tendency to inflammatory condition in the tissues about a cast denture than with a swaged denture, observes Dr. Jones. This, he thinks, may be due to the copper coming to the surface of the cast metal and so getting into intimate contact with the tissues.

The author finds that ''in the application of cast dentures to lower cases, we get our best results, for once the ridge has set we get a uniform hardness of tissue and process to receive a like uniform stiff structure. In lower cast dentures, if the cast is carried hard up on the necks of the teeth, I have found that the teeth either move with constant pressure or the tissue surrounding the necks of the teeth is pushed back; this may occur in swaged plates, but not to the same extent. To avoid this I have always adopted, with beneficial results, the plan of slightly easing the cast around the necks of the lower teeth.''

Another point of interest is cited by Dr. Jones. Remarkable results, he says, were produced in casting partial dentures (upper) upon impressions taken in modeling compound. He refers to two cases—one carrying six teeth, another eight—in which ''the fit today is better than when inserted four years ago; in both cases the tissue was extremely spongy and the teeth elongated.'' ''The way I account for this,'' he says, ''is that the softness of the tissue has given the rigidity of the plate its natural spring and seat.

INVESTING AND CASTING GOLD INLAYS

In the *Journal of Allied Societies*, Dr. Conzett writes about casting and investing methods. He first of all suggests that we prepare our own investing material, the formula for which is as follows: One part of plaster of Paris, three parts of finely pulverized silex and one-half part of Dixon's flake graphite. These constituents are mixed together thoroughly and then passed through an ordinary flour sifter three or four times. This preparation, the author claims, can ''be made a good deal more cheaply than bought.''

In preparing a mix for investment, the same quantities, by measure, of water and investment material are used. ''We are aiming to keep all of the processes at room temperature, so we use water that is about one hundred to one hundred and twenty degrees in temperature, according to the temperature of the room, for if we use water that is just the right temperature we shall find that the cold bowl, cold plaster, etc., will make the investment too cold, while the water being a little warmer than necessary will obtain a mixture that is just about right without the bother of complex paraphernalia to obtain the same result.''

Preparatory to applying the investment material, the model is washed off with a little soap on a camel-hair brush. The idea of this is to remove any material, such as oils, etc., that may interfere with the perfect adaptation of the investment material. "The model is now coated with the soft investment material, carefully excluding any bubbles and the investment completed in the flask. As soon as the investment is hard I begin to burn out, and am very careful that this is not overdone. More inlays are spoiled by reason of the overheating of the investment than any other way, for the plaster is disintegrated and made weak by too much heat, and under pressure of casting, gives ground to the force of pressure and a distorted misfitting inlay is the result. I have recently installed a one-heat electric heater that is giving the greatest satisfaction. I leave the moist flask—that is, before the moisture has been driven off, on the heater that has had the current on for about a minute and then turned off, for five minutes, when I know that the investment is dry and ready to heat. The current is then turned on and left for ten minutes, after which the current is turned off and the flask set to cool."

Casting is done into a cold flask. The gold is melted with the oxy-hydrogen blow-pipe because of its quickness and the effect produced. The use of the ordinary blow-pipe would result in the heating up of the entire flask, and this is not desired.

Gold of 23k, is used where the inlay is subject to masticatory stress, and in all other situations pure gold is used.

If pressure casting is to be used then a force of five pounds is sufficient for the purpose and should be maintained for at least thirty seconds. Before casting, the gold is brought to a white heat and not allowed to boil.

After removing the inlay from the flask it is washed and pickled in hydroflouric acid for a few minutes to remove all traces of calcareous material.—*Oral Health.*

